# The potential role of mitochondrial ATP synthase inhibitory factor 1 (IF1) in coronary heart disease: a literature review

**DOI:** 10.1186/s12944-017-0430-9

**Published:** 2017-02-07

**Authors:** Serban Maierean, Maria-Corina Serban, Manfredi Rizzo, Giuseppe Lippi, Amirhossein Sahebkar, Maciej Banach

**Affiliations:** 1 Victor Babes University of Medicine and Pharmacy, Timisoara, Romania; 2 Department of Functional Sciences, Discipline of Pathophysiology, “Victor Babes” University of Medicine and Pharmacy, Timisoara, Romania; 3 Biomedical Department of Internal Medicine and Medical Specialties, University of Palermo, Palermo, Italy; 4 Euro-Mediterranean Institute of Science and Technology, Palermo, Italy; 5 Section of Clinical Biochemistry, University of Verona, Verona, Italy; 6 Biotechnology Research Center, Mashhad University of Medical Sciences, Mashhad, Iran; 7 Metabolic Research Centre, Royal Perth Hospital, School of Medicine and Pharmacology, University of Western Australia, Perth, Australia; 8 Department of Hypertension, WAM University Hospital in Lodz, Medical University of Lodz, Zeromskiego 113, 90-549 Lodz, Poland; 9 Polish Mother’s Memorial Hospital Research Institute, Lodz, Poland

**Keywords:** Angiogenesis, Cardiovascular disease, High density lipoprotein, Inhibitory factor 1

## Abstract

Cardiovascular disease (CVD) is the leading cause of death worldwide, and so the search for innovative and accurate biomarkers for guiding prevention, diagnosis, and treatment is a valuable clinical and economic endeavor. Due to a recent findings that the serum concentration of mitochondrial ATP synthase inhibitory factor 1 (IF1) is an independent prognostic factor in patients with coronary heart disease (CHD), we reviewed the role of this protein in myocardial ischemic preconditioning, its correlation to plasma high density lipoprotein (HDL), the predictive potential in patients with CHD, and its interplay with angiogenesis. IF1 has been positively correlated with plasma HDL-cholesterol, and is independently negatively associated with all-cause and CV mortality in patients with CHD. However, this conclusion is prevalently based on limited data, and more research is needed to draw definitive conclusions. IF1 seems to play an additional role in increasing cell vulnerability in oncologic diseases but may also function as modest inhibitor of angiogenesis in physiological conditions. It has been also explored that IF1 may rather act as a modulator of other molecules more significantly involved in angiogenesis, especially apolipoprotein A1 on which the largest effect could be observed. In conclusion, more research is needed to characterize the role of IF1 in patients with CHD.

## Background

Cardiovascular disease (CVD) is the leading cause of death worldwide, therefore the search for sensitive biomarkers for CVD prevention, diagnosis, and treatment seems to be still critical [1–3]. Mitochondrial ATP synthase, also referred to in some literature as F_1_F_0_-ATPase, is the 5^th^ complex of the mitochondrial electron transport chain, the function of which entails utilization of proton motive force (PMF) established by the previous complexes in order to catalyze the energetically-unfavorable ATP synthesis [4]. Under hypoxic conditions, the PMF dissipates and is unable to propel the activity of ATP synthase. This leads to a shift in enzyme function, catalyzing the more energetically favorable hydrolysis of ATP [4]. In 1995, Vuorinen et al. first investigated the role of F1F0-ATPase in preconditioning of rats’ hearts to ischemia, demonstrating that the inhibition of this enzyme led to release of cytosolic ATP and to the improvement of time-averaged energy state of the heart during subsequent episodes of prolonged ischemia [5]. A successive study convincingly suggested that mitochondrial ATP synthase inhibitory factor 1 (IF1) is the molecule responsible for ischemic preconditioning (IP) by inhibiting the F1 subunit of the F1F0 complex during hypoxic states [6]. It was also noticed that larger mammals, especially those with slower heartbeats, experienced a more pronounced release of ATP due to hydrolysis [6]. Further investigation revealed that up to 80% of cytosolic ATP hydrolyzed during ischemic episodes in some species can be attributed to the activity of F1F0-ATPase alone [7], and that overexpression of IF1 protected from ischemic damage [8]. After discovery that mRNA, which is transcribed into the *beta*-chain of the F1 subunit of the F1F0-ATPase (the site of action of IF1), is transcriptionally active in hepatocytes [9], Martinez et al. demonstrated that such ectopic *beta*-chains are found on cell surface of hepatocytes, and that they act as apolipoprotein A-I (apoA-I) receptors, thus mediating holo-endocytosis of high-density lipoprotein cholesterol (HDL-C) [10]. This finding paved the way to uncover many potential clinical implications, such as the possible use of these ectopic *beta*-chains as potential drug targets in patients with, or at risk of developing coronary heart disease (CHD). In light of these findings, Genoux et al. [11] performed a cross-sectional study for evaluating the correlation between serum concentrations of exogenous ecto-F1-ATPase inhibitor, IF1, HDL-C and triglycerides. It was hence observed that serum IF1 concentrations were directly correlated with HDL-C values and negatively associated with triglycerides [11]. Subsequent research by the same team of scientists confirmed that serum IF1 concentration is positively associated with HDL-C concentrations and negatively with CHD [12], and finally that IF1 has an independent and positive association with long-term prognosis of CHD [13]. These findings bring about many potential implications in prevention of CHD and management of patients with established disease. IF1 has also been studied in the context of angiogenesis [14–16]. Provided that IF1 interplays with angiogenesis, a relationship may also be put forward between the number of collateral arteries in the hearts of patients with different baseline IF1 values [17].

The purpose of this review is to aggregate and critically analyze the areas where IF1 might be implicated in cardiovascular (CV) health, to assemble the bulk of knowledge currently available on this topic, and discuss areas where future research would be of great interest and importance for increasing the knowledge about the vast potential of IF1. This review will hence focus on the role of IF1 in curbing cellular ischemic injury, on its relationship with HDL-C, its predictive potential in CHD and its potential interplay with angiogenesis.

## Search strategy

We carried out an electronic search on electronic scientific databases [MEDLINE (1966-14^th^ August 2016), EMBASE and SCOPUS (1965-14^th^ August 2016), DARE (1966-14^th^ August 2016)], and Web of Science Core Collection (up to 14^th^ August 2016). Abstracts from national and international meetings were also searched. When necessary, the relevant authors were contacted to obtain additional information. The main search terms were: angiogenesis, atherosclerosis, arteriosclerosis, endothelial dysfunction, cardiovascular, cardiovascular disease, coronary heart disease, CHD, CVD, high density lipoprotein, inhibitory factor 1, ischemic, mitochondrial ATP synthase. The main inclusion criterion was the existence of relatively strong data from studies, trials and meta-analyses investigating the role of IF1 in lipid disorders, angiogenesis and cardiovascular function.

## Ischemic cellular damage and myocardial ischemic preconditioning

One of the more investigated roles of IF1 is its capability to reduce ATP hydrolysis when the cell is challenged by hypoxia [7]. The binding of IF1 to ATP synthase is promoted by a decrease in both membrane potential and mitochondrial matrix pH [7]. It is now clear that IF1 is directly implicated in mitigating the generation of ADP from ATP by means of hydrolysis, as reflected by the relationship between ATP production and IF1 concentration, as well as by its species-specific affinity for the beta-subunit of F1 [6]. In order to verify whether IF1 may directly inhibit the hydrolysis of ATP mediated by ATP synthase, Rouslin et al. [6] pre-treated animals’ hearts with the uncoupling agent carbonyl cyanide p-trifluoromethoxyphenulhyobrozone (FCCP), which acts through dissolution of the PMF. Their most relevant finding was that the rabbit cardiomyocytes, whose mitochondria housed relatively higher concentrations of high-affinity IF1, were more effective at preserving ATP during short bouts of ischemia than rat and pigeon hearts, whose cardiomyocytes contained lower levels of high-affinity IF1 and a full complement of lower-affinity IF1, respectively [6]. However, after 30 min of ischemia, all pre-treated hearts contained almost undetectable concentrations of ATP [6]. This evidence is supported by research showing that HeLa cells with permanent IF1 knockout have a ~30% lower concentration of ATP under ideal growing conditions compared to non-knockout cells [18]. This seems to suggest that antagonism of ATP hydrolysis by IF1 not only functions when the cell is challenged by ischemia, but also under physiological conditions [18]. However, when exposed to ischemia under normal conditions (i.e., not under the effects of uncoupling agents), Hassein et al. put forward the hypothesis that the bulk of observed beneficial effects of IF1 are maybe not directly due to inhibition of enzymatic activity of ATP synthase, but rather to inhibition of proton conductivity of ATP synthase [7] achieved by physically blocking ATP synthase’s H + −channel [19]. Preserving the proton electrochemical gradient in the mitochondrial membrane inhibits the reversal of ATP synthase, and also limits reperfusion damage [7]. They also suggested that IF1 may play a role in increasing the coupling efficiency of the complex [20], as in Luft’s disease, which is characterized by mitochondrial uncoupling [7]. By limiting reperfusion damage and helping maintain the PMF, IF1 could potentially be implicated in post-ischemic recovery, although this remains to be further elucidated [7].

Regardless of the specific mechanism of action of IF1 in reducing ischemic injury, Genoux et al. prospectively studied 577 males with stable CHD for a median follow-up of 11 years, and observed that patients with CHD who died had lower serum concentration of IF1 and higher values of N-terminal pro-brain natriuretic peptide (NT-proBNP) and high sensitivity troponin T (hsTnT) (i.e., two biomarkers associated with myocardial injury) compared to those who survived [13].

Interestingly, another research conducted by Fujikawa et al. showed that mitochondria of HeLa cells with a permanent IF1 knockout seemingly generate greater PMF and produce almost double the amount of reactive oxygen species (ROS) as control cells under ideal growth conditions (i.e. not under ischemic stress) [18], a finding that was then confirmed in a separate research [21]. These results should raise interest in the possibility of increased baseline cellular turnover in persons who express lower levels of IF1, secondary to mitochondrially-induced apoptosis due to the increase in local ROS.

Despite many findings relating to the involvement of IF1 in releasing ATP during ischemia, its role in myocardial ischemic preconditioning remains controversial. In 1990, Das and Harris proposed that the bidirectional functionality of ATP synthase is modulated by a regulatory protein [22]. Afterwards, another study reported that preconditioned rat hearts displayed a sustained ATP synthase inhibition, thus benefiting the rats upon subsequent ischemic challenges [5]. Opposite results were obtained in another study focused on inhibition of F1F0-ATPase of canine hearts, which showed that although energy sparing is a feature of ischemic preconditioning, ATPase inhibition is not the most likely explanation for this phenomenon [23]. Yet a subsequent study was carried out, reporting that the baseline activity of F1F0-ATPase was not significantly different in preconditioned versus control mitochondria, but the time required for the enzyme to reach steady-state was increased in the preconditioned mitochondria before sustained ischemia [24]. The authors concluded that if ATP synthase is supposed to play a role in ischemic precondition, this might only be modest [24]. Therefore, the exact role of IF1 in ischemic preconditioning remains controversial. More clinical potential exists in understanding the extent to which IF1 can help patients vulnerable to ischemic injury, especially those at higher risk of developing a myocardial infarction.

## Correlation between IF1, HDL and CHD

**Fig. 1:**
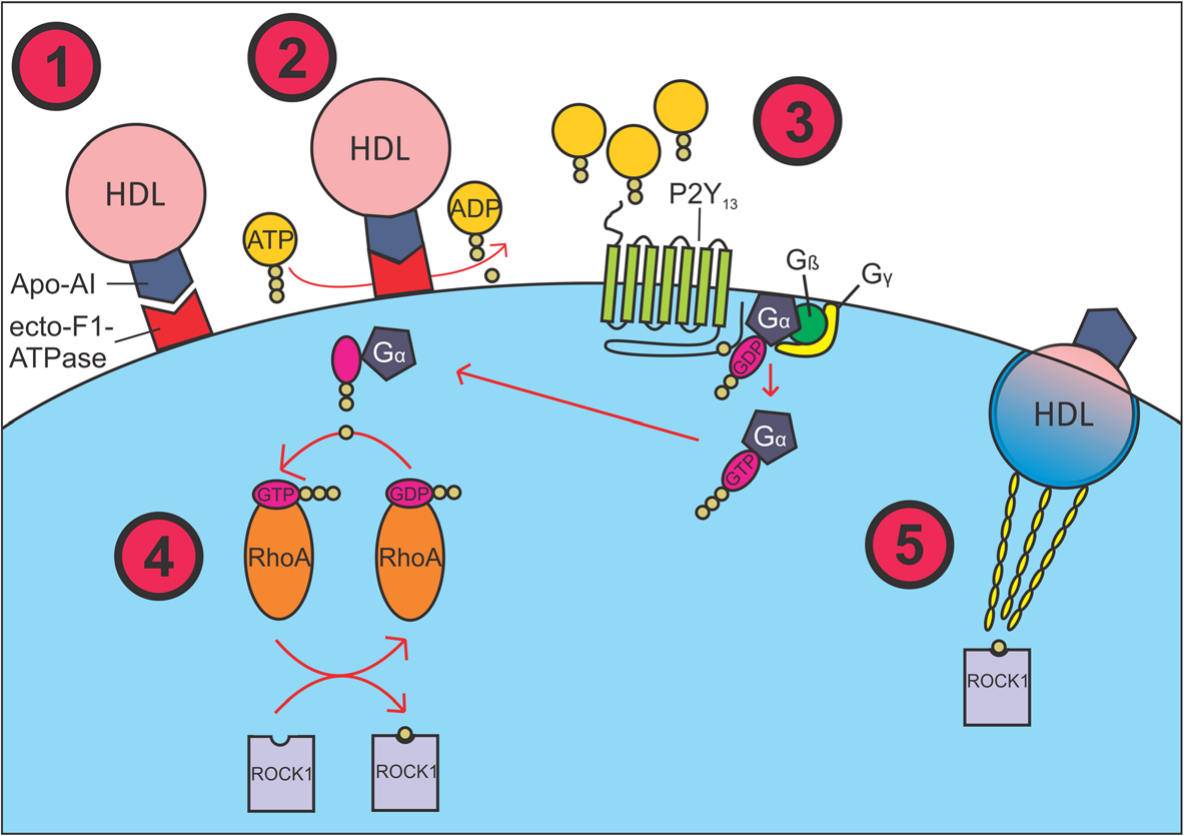
The pathway of ecto-F1-ATPase-mediated holo-HDL endocytosis. (1) ApoA-I, found on the HDL molecule, binds to the beta-subunit of the ecto-F1-ATPase of a hepatocyte; (2) This binding activates ecto-F1-ATPase, causing it to increase the production of ADP from ATP; (3) With the extracellular concentration of ADP increased, P2Y_13_, a G protein-coupled receptor, is activated, phosphorylating the GDP molecule found on the alpha-subunit of the G Protein. This phosphorylation activates the G protein, releasing it from its beta and gamma subunits; (4) The G Protein then activates RhoA by phosphorylation, causing the G Protein to become inactive where it will return to join its beta and gamma subunits on the inner plasma membrane. Activated RhoA will then donate a phosphate group to ROCK1, activating the protein; (5) ROCK1 then drives the cytoskeletal modifications necessary for holo-HDL endocytosis

Despite the fact that F1F0-ATPase is a mitochondrial protein, the F1 catalytic subunit of this enzyme was found to be also present on the plasma membrane of rat hepatocytes [9], herein conventionally referred to as ecto-F1-ATPase. Interestingly, ecto-F1-ATPase was shown to mediate holo-HDL endocytosis in hepatocytes via the interaction between the *beta*-subunit of F1 and apoA-I of HDL particles [10], a distinct pathway from that entailing the more important scavenger receptor-B1 HDL-uptake, according to which the protein components of HDL are not endocytosed [25]. Martinez et al. have recently explained this pathway to function roughly as follows [26] (Fig. 1): (1) the apoA-I protein moiety of an HDL particle binds to the beta-subunit of ecto-F1-ATPase; (2) extracellular ADP, which is normally kept in balance by ecto-F1-ATPase and adenylate cyclase, increases by activation of ecto-F1-ATPase; (3) the increased concentration of extracellular ADP activates P2Y_13_-receptor, a G-protein coupled receptor; (4) in turn, this activates the guanosine triphosphatase (GTPase) *RhoA* via phosphorylation, which then activates *Rho*-associated kinase I (ROCK I), also via phosphorylation; (5) the cell finally undergoes cytoskeletal reorganization, thus resulting in endocytosis of HDL [26].

The inhibition of ecto-F1-ATPase seems to only affect HDL uptake, with no effects on the transport of LDL [26]. Unlike in myocardial cells, IF1 binding to F1 is not improved by decreased pH [26]. This may suggest that the F0 subunit of the F1F0-ATPase, which is missing on cellular plasma membranes [10], might perhaps be the determinant of whether the complex is used for HDL transport or cell survival during hypoxic conditions, although this remains a mere speculation. It has also been observed that ecto-F1-ATPase is present on the surface of endothelial cells where, along with P2Y_12_, mediates HDL transmigration. Although the physiological and clinical significance of this mechanism is still unclear, it seems reasonable to suggest that ecto-F1-ATPase activation might play a role in either accumulation of HDL in foam cells and enhancement of reverse cholesterol transport (RCT) [26]. Inhibition of ecto-F1-ATPase by IF1 should then result in an increase in total circulating HDL-C by reducing its endothelial uptake. Although a strong correlation exists between IF1 and HDL-C [11–13], the existence of a causal relationship has not been definitely proven; neither is clear whether or not increased HDL resulting from exogenous ecto-F1-ATPase inhibition (i.e. via pharmacotherapy) would finally translate into significant clinical advantages. Perhaps, while IF1 increases plasmatic HDL concentrations by reducing its hepatic uptake, inhibition of the same receptors at the endothelial level might also reduce the degree of HDL transmigration, which might reduce the benefits of the increased plasma HDL concentration.

From a clinical perspective, Genoux et al. published a series of studies aimed to investigate the association between serum IF1, plasma HDL, and their correlation with the prevalence of CHD and patient survival. Interestingly, the serum concentration of IF1 (medians of 0.43 ± 0.13 mg/L, *p* < 0.001 [cases], and 0.53 ± 0.15 mg/L, *p* < 0.001 [controls]) was found to be positively associated with plasma HDL-C (r = 0.27, *p* < 0.001) and apoA-I (r = 0.28, *p* < 0.001) and negatively with triglycerides (r = −0.23, *p* < 0.001) (case-control study with 648 cases and 669 controls, males) [12]. These findings were then confirmed by measurements carried out in previous (cross-sectional study with 100 normolipidemic male participants) [11] and later (prospective cohort with 577 male participants suffering fromcoronary artery disease [CAD]) [13] studies by the same team of authors. The mean serum IF1 concentration was also found to be lower in patients with CHD (0.43 mg/L, *p* < 0.001) than in control subjects without CHD (0.53 mg/L, *p* < 0.001), and was significantly correlated with CHD after adjusting for plasma HDL and apoA-I (*p* < 0.001) [12]. A higher CHD risk reduction was noticed in patients with low HDL and high IF1 (69% lower odds ratio (OR = 0.31, 95% CI [0.20, 0.48], *p* < 0.001)) than in patients with median values of HDL and IF1. Patients with high HDL and low IF1 also exhibited a reduced risk of CHD (OR = 0.24, 95% CI [0.15, 0.37], p < 0.02). Expectedly, the presence of elevated concentrations of both HDL and IF1 conferred an even larger risk reduction (OR = 0.14, 95% CI [0.09, 0.21], *p* < 0.001); patients with low values of apoA-I and high concentration of IF1 had a comparable risk reduction (OR = 0.32, 95% CI [0.20, 0.51], *p* < 0.001), whereas those with elevated values of both had the greatest risk reduction (OR = 0.10, 95% CI [0.07, 0.15], *p* < 0.001). However, patients with only apoA-I elevated displayed a similar risk [OR = 0.14, 95% CI [0.09, 0.21], p < 0.001) [12]. The finding that increased apoA-I conferred a similar risk reduction of CHD as both elevated apoA-I and IF1, while the same did not hold true for HDL and IF1, is interesting and merits further investigation and confirmation. As IF1 is a competitive antagonist of apoA-I [10], it seems reasonable to suggest that a high apoA-I concentration may reflect an enhanced production to compensate for a high baseline IF1 value, so not translating to improved outcomes. This hypothesis remains to be investigated, however.

In their most recent research, Genoux et al. measured serum IF1 concentration in 577 males with stable CHD, and found that both cardiovascular and all-cause mortality were lower for patients in the highest quartile of IF1 (hazard ratio [HR] 0.50, 95%CI 0.28–0.89, and 0.55, 95%CI 0.38–0.89, respectively), whereas death rate was virtually identical across quartiles of HDL [13]. While surprising, this finding had been confirmed by additional research showing that only the large HDL subfraction (measured using the Lipoprint® system) [27], but not total HDL, is associated with decreased mortality in patients with CHD [27, 28]. Unlike HDL, IF1 does not seem to be related to blood pressure, nor with alcohol consumption [13], which slightly differentiates it from traditional cardiovascular risk factors. Contrarily, IF1 was found to be positively correlated with left ventricular ejection fraction (LVEF) and negatively associated with severity of CAD) [13]. Overall, IF1 seems a better marker for predicting outcomes in patients with CHD than total HDL, although larger prospective studies may be needed to validate this conclusion [29]. Moreover, additional research should be planned to explore the relationship between IF1 and other risk factors of CVD, especially physical activity, body mass index and waist circumference. Studies of homozygous versus heterozygous twins would also be beneficial in identifying the most significant determinants of IF1 concentration. Finally, although higher IF1 values correlate with a lower risk of CHD and better outcomes in patients with CHD, it remains unclear whether therapeutically driven enhancement of IF1 concentration may generate comparable outcomes.

## IF1 and angiogenesis

From a physiological perspective, evidence was brought that IF1 may act as a weak inhibitor of angiogenesis, whereas a more effective role has been shown in protecting endothelial cells from hypoxic damage, thus mirroring its function in mitochondria [15]. Burwick et al. compared the effect on angiogenesis in vitro on human umbilical vein endothelial cells (HUVEC) of IF1, angiostatin and cycloheximide [15]. An IF1 concentration of 10 μg/mL was found to have a 20% inhibitory effect on endothelial cell proliferation, whereas inhibition was 60% using cycloheximide, a well-known proliferation inhibitor [15]. Earlier results from the same team of scientists reported that angiostatin was able to inhibit endothelial cell proliferation by 57% [30]. Although IF1 appears to possess a certain inhibitory potential on endothelial cell proliferation, its modulatory capacity should be considered globally modest. In fact, unlike cycloheximide, IF1 is not effective in inhibiting endothelial cell tube formation [15]. Interestingly, Burwick et al. noticed that angiostatin competes with and thereby antagonizes IF1 for the active site of cell surface ATP synthase, ultimately reducing its ability to conserve ATP at low pH. Whether angiostatin and IF1 may be competitive inhibitors remains to be elucidated [15]. It was hence hypothesized that IF1 may be a protective factor for endothelial cells under condition of low pH such as in tumor microenvironment, and that inhibiting ATP hydrolysis alone may be insufficient for preventing angiogenesis, which actually requires inhibition of ATP synthesis [15].

Despite available evidence that IF1 might not be a significant modulator of angiogenesis, at least directly, the results of a recent study suggest that it is instead an antagonist to apoA-I, which was found to promote angiogenesis in human endothelial progenitor cells (hEPC) [16]. This study evidenced that hEPCs express ATP synthase on the surface of their plasma membrane, and like in hepatocytes, these cells bind apoA-I. It was also observed that 50 μg/mL of apoA-I was a sufficient concentration to enhance cellular proliferation by 14.5% (*p* < 0.001), and the number of branch points and length of tubules by 31% (*p* < 0.01) compared to controls [16]. The addition of IF1 and of the known inhibitor oligomycin separately, completely inhibited these effects, whereas inhibition of the scavenger receptor class B type I (SR-BI) alone only partially reduced the angiogenic effects of apoA–I [16].

From a clinical perspective, recent evidence was brought that overexpression of IF1 in hepatocellular carcinoma (HCC) is a poor prognostic indicator, and that this cancer displays a larger degree of angiogenesis, more pronounced active epithelial-mesenchymal transition, and more frequent metastasis than HCC tumors that express normal levels of IF1 [14]. Silencing of IF1 expression resulted in the attenuation of epithelial-mesenchymal transition and invasion of HCC cells [14]. The likely biochemical pathway to explain these observations entails that IF1 may promote overexpression of Snail (a Zinc-Finger transcription factor that leads to a downregulation of E-cadherin [31], allowing angiogenesis during embryonic development ultimately mediated by epithelial-mesenchymal transition [32] and of vascular endothelial growth factor (VEGF) through nuclear factor-kappa B (NF-κB) signalling. NF-κB is then thought to generate a positive feedback loop with IF1 by direct upregulation of the protein [14].

In conclusion, despite the evidence that IF1 might be somehow involved in the modulation of angiogenesis, additional research is needed to objectively confirm these preliminary findings. Based on data presented in this review, it seems reasonable to conclude that IF1 may only promote angiogenesis in neoplastic conditions [14]. It is possible, however, that it may act as an indirect modulator of angiogenesis by interplaying with other proteins such as apoA-I [16].

## Conclusions

Although IF1 does not apparently play a key role in myocardial ischemic preconditioning, the available evidence suggests that it may be effective in limiting cellular damage during short bouts of ischemia and reperfusion. Additional research is hence needed to better understand the relationship between blood IF1 concentration and outcomes in acute coronary events. In patients with stable CHD, higher concentrations of serum IF1 seem to be associated with better outcomes, but new studies should be planned to verify whether a therapeutically driven increase of serum IF1 may generate comparable (favorable) outcomes in patients with stable CHD. Regarding the involvement of IF1 in angiogenesis and cardiovascular health, again no definitive conclusions can be drawn, mainly due to the heterogeneity of the available studies. Nevertheless, IF1 seems to be involved in both promoting and inhibiting angiogenesis, in different contexts.
